# Blood-Corpuscles of the Stanley Musk Deer

**Published:** 1843-07

**Authors:** 


### Blood-corpuscles of the Stanley Musk Deer.

"The average diameter of the blood-discs of the Stanley musk-deer, (*Moschus Stanleyanus,* Gray) is $\frac{1}{10825}$th of an English inch. Hence they are nearly as small as those of the Napu musk-deer, (*Moschus Javanicus,* Pallas.)" (Abstract of a paper read by Mr. Gulliver at the Zoological Society of London, May 9th, 1843.)

In the British and Foreign Medical Review, Vol. XIV. p. 570, will be found some measurements of the smallest blood-corpuscles of mammals, from which it appears, according to Mr. Gulliver's observations, that the blood-discs of the Napu musk-deer and of the ibex, (*Capra Caucasica,*) are minuter than any before described. The blood-discs of the Stanley musk-deer are intermediate in size to those of the Napu musk-deer and ibex, and therefore much smaller than the corpuscles of the common goat, (*Capra Hircus.*)

